# Should I Stay or Should I Go? Dispersal and Population Structure in Small, Isolated Desert Populations of West African Crocodiles

**DOI:** 10.1371/journal.pone.0094626

**Published:** 2014-04-16

**Authors:** Guillermo Velo-Antón, Raquel Godinho, João Carlos Campos, José Carlos Brito

**Affiliations:** 1 CIBIO/InBIO, Centro de Investigação em Biodiversidade e Recursos Genéticos da Universidade do Porto, Instituto de Ciências Agrárias de Vairão, Vairão, Portugal; 2 Departamento de Biología da Faculdade de Ciências da Universidade do Porto, Porto, Portugal; State Natural History Museum, Germany

## Abstract

The maintenance of both spatial and genetic connectivity is paramount to the long-term persistence of small, isolated populations living in environments with extreme climates. We aim to identify the distribution of genetic diversity and assess population sub-structuring and dispersal across dwarfed desert populations of *Crocodylus suchus*, which occur in isolated groups, usually less than five individuals, along the mountains of Mauritania (West Africa). We used both invasive and non-invasive sampling methods and a combination of mitochondrial DNA (12 S and ND4) and microsatellite markers (32 loci and a subset of 12 loci). Our results showed high genetic differentiation and geographic structure in Mauritanian populations of *C. suchus*. We identified a metapopulation system acting within four river sub-basins (high gene flow and absence of genetic structure) and considerable genetic differentiation between sub-basins (*F*_ST_ range: 0.12–0.24) with rare dispersal events. Effective population sizes tend to be low within sub-basins while genetic diversity is maintained. Our study suggests that hydrographic networks (temporal connections along seasonal rivers during rainy periods) allow *C. suchus* to disperse and maintain metapopulation dynamics within sub-basins, which attenuate the loss of genetic diversity and the risk of extinction. We highlight the need of hydrographic conservation to protect vulnerable crocodiles isolated in small water bodies. We propose *C. suchus* as an umbrella species in Mauritania based on ecological affinities shared with other water-dependent species in desert environments.

## Introduction

Habitat connectivity is important for populations living in climate-extreme environments, which are naturally patchy and have temporally variable resources [1]. In particular, water-dependent species in desert environments are examples for dispersal limitations owing to scarce and unpredictable availability of water sources, primary production and shelters [2]. The migration movements of such species are usually constrained by extensive barriers, thus they disperse mostly through a limited number of natural corridors such as hydrographic basins. These basins represent important passageways for the dispersal of numerous relictual aquatic species characterized by small and fragmented distributions [3]. In the Sahara, waterways played a crucial role in the historical dispersal of organisms and still may function as contemporary dispersal routes for several taxa with distinct biogeographical origins (e.g. Nile river) [4], [5]. The current status of relict *Crocodylus suchus* [6] populations across the Sahara-Sahel exemplifies the threats associated with loss of landscape connectivity for aquatic vertebrates with low population densities and restricted dispersal abilities [5]. This species was historically widespread over North Africa but was extirpated throughout much of its original distribution. Increasing aridification in the Sahara-Sahel since the Mid-Holocene (7000 yr) gradually dried the savannah-like ecosystem and most aquatic habitats, which induced local extinctions of aquatic/amphibious taxa [5], [7]. Further, anthropogenic pressures in the first half of the last century left only a few relict populations in Chad and Mauritania [8], [9].

In Mauritania, crocodile populations are known from 81 small rocky pools (locally known as *gueltas*), located upstream of mountain valleys, and floodplains (locally known as *tâmoûrts*), located at the foothills of the mountains [8], [9]. The small area of *gueltas* (0.001–1.0 ha) restricts their carrying capacity, thus they usually harbour less than five adult individuals [8]. Crocodiles inhabiting these features are highly vulnerable to demographic and environmental variability, which in turn may reduce their genetic diversity and increase risk to their adaptive capacity due to stochastic changes [10], [11]. However, during the rainy season (July to October), dispersal corridors are formed along raging streams that usually flow to vast plains adjacent to the *gueltas* and *tâmoûrts* [8], [9]. These periodical and short-timed events can seasonally connect isolated habitats, eventually allowing the rescue effect of small populations [8]. Field surveys across Mauritanian mountains have detected possible individual dispersal between water bodies (usually less than 4 km) during the rainy season [8], suggesting that populations of *C. suchus* may constitute a metapopulation. Analyses of molecular markers are needed to corroborate field observations and interpretations about the potential connectivity, possibly enabling gene flow between crocodile populations and confirming the existence of a dynamical metapopulation. Assessing genetic structure and gene flow among populations, as well as identifying landscape features that have an impact on gene flow are key to identify dispersal corridors as targets for conservation efforts. The genetic patterns of these relict crocodile populations may help understand possible metapopulations dynamics in desert landscapes and contribute to the prioritization of conservation actions on a species that could serve as a model for connectivity conservation, i.e. strategies for protecting and restoring dispersal corridors [12], [13].

Using a combination of mitochondrial DNA (mtDNA) and microsatellite markers, we aim to: 1) assess population sub-structuring across isolated populations of *C. suchus* in Mauritania; 2) detect the occurrence of contemporary gene flow among *gueltas*/*tâmoûrts* and between sub-basins; and 3) quantify genetic diversity within each sub-basin. Taking into account isolated populations and small group size within each locality; we predict genetic structuring, reduced gene flow, low levels of genetic diversity and signs of inbreeding. However, the dispersal ability of crocodiles during the rainy season might counteract the negative effects of their isolation.

## Methods

### Ethics statement

Fieldwork was developed with permission from the Ministére Délégué auprès du Premier Ministre Chargé de l'Environnement, Nouakchott (Permit: 460/MDE/PNBA). This permit was valid for the entire country and no specific permissions were required for any specific locality. Analyses were done at a CITES registered laboratory: 13PT0065/S. Field collection and handling practices were approved by the Committee of Animal Experimentation of the University of Porto (Portugal) under the Directive 2010/63/EU of the European Parliament. No animal was sacrificed and there were no animal husbandry, experimentation and care/welfare concerns.

### Study area and sample collection

**Figure 1:**
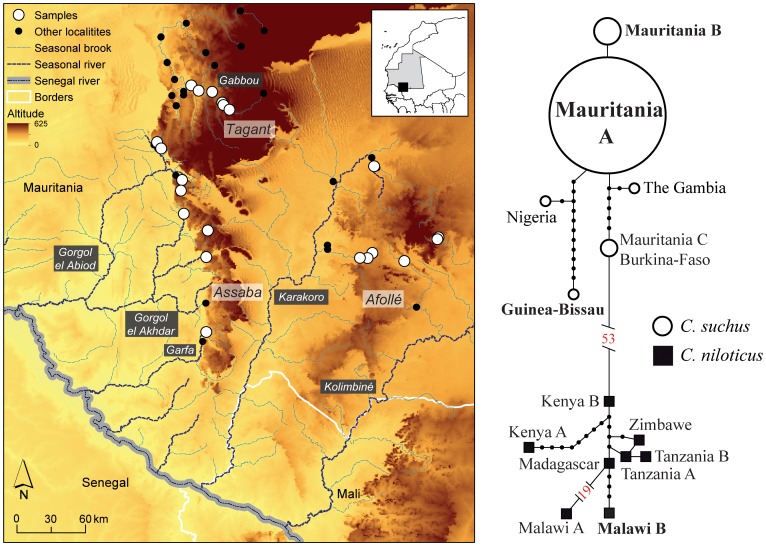
On the left: Distribution of *Crocodylus suchus* localities along major river sub-basins (Gabbou, Gorgol el Abiod, Gorgol el Akhdar-Garfa and Karakoro-Kolimbiné) of southern Mauritania mountains (Tagant, Assaba and Afollé). White dots represent sampled localities for the current genetic study, whereas black dots represent other localities where crocodiles are currently present (Adapted from Brito et al. 2011a). On the right: Haplotype network based on mtDNA (12 s+ND4 markers). White circles represent *C. suchus* individuals while black squares indicate *C. niloticus* individuals. In bold, haplotypes that represent individuals sampled in this study. Numbers indicate the number of mutational steps between independent networks.

The study area encompasses three southern Mauritanian mountains: Tagant, Assaba and Afollé (Figure 1). These mountains are associated with six important seasonal hydrographic sub-basins [14]: 1) the endorheic Gabbou that flows within the Tagant, and is isolated and composed by several permanent and inter-connected *gueltas*; 2) the Gorgol el Abiod that results from water draining from the southern Tagant and northern Assaba; 3) the Gorgol el Akhdar and 4) Garfa sub-basins that are formed through water run-offs from the western Assaba; 5) the Karakoro that flows between the Assaba and the Afollé, although it is mostly fed by run-offs from the later mountain, and is characterized by low water availability; and 6) the Kolimbiné that flows east-ward of the Afollé. With the exception of the Gabbou, the other five sub-basins flow to the Senegal River and are composed of *gueltas* in mountain slopes and seasonal *tâmoûrts* at foothills of the mountains [14]. Climate is characterized by a dry and cool season from November to February and a dry, hot season from March to June [15]. Annual rains are scarce and seasonal, occurring in a single wet period from July to October, with most precipitation in August and September [15]. The area is remote and devoid of anthropogenic infrastructure and was recently affected by regional conflict caused by political instability. These factors sometimes hamper field surveys and trans-border research and conservation planning [16].

We collected a total of 94 samples of *C. suchus* from fieldwork expeditions from 2007 to 2011 [8], [9]. Samples include 16 fresh tissues, nine near fresh tissues (from specimens found dead), four skin fragments from carcasses, and 65 faecal remains (Fig. 1). The samples collected represent *ca*. 25% and 5% of the total population of crocodiles in the Tagant/Assaba and Affolé mountains respectively. We captured live crocodiles by hand or using hand nets, and then immediately released them at the point of capture after clipping a 5 mm piece of tail tissue for genetic analysis. Team members obtained the tissue samples following ethical guidelines for use of live reptiles. We used samples obtained from carcasses of *C. suchus* from Guinea-Bissau and *C. niloticus* from Malawi as outgroups in the analyses (see Table S1).

### DNA extraction, sequencing and genotyping

We performed DNA extractions for fresh and near-fresh tissues using the EasySpin Genomic DNA Tissue Kit or the QIAamp DNA Micro Kit (QIAGEN). We followed Frantz et al., [17] protocol after the GuSCN/silica method [18] for DNA extractions of faecal remains, while used QIAamp DNA Micro Kit (QIAGEN) for skins. We performed all pre-PCR procedures of non-invasive samples in a dedicated laboratory used only for the manipulation of low quality DNA under sterile conditions and quantified DNA through fluorimetry, excluding samples with a DNA concentration lower than 0.4 µg/ml.

We amplified two mtDNA fragments of 421 bp and 878 bp from 12 S and ND4, respectively, for fresh and near fresh samples in 10 µl reaction volumes, containing 5 µl of master mix (Multiplex PCR Kit, QIAGEN), 0.5 µM of each primer and approximately 1 µl of genomic DNA (see Table S2 for primers and PCR conditions). We performed cycle sequencing for both strands using PCR primers and the ABI PRISM BigDye Terminator kit (AB Applied Biosystems) in a My Cycler BioRad Thermal Cycler and sequenced PCR products on an ABI 3130xl Genetic Analyzer (AB Applied Biosystems).

We genotyped invasive samples for a total of 32 microsatellite loci previously developed in crocodilian species [19], [20] and used a subset of 12 loci to genotype non-invasive samples due to their Probability of Identity (PID) power and small fragment sizes (see Table S2 for description of markers, primers, allele range and PCR conditions of each multiplex reaction). The final genotype database comprises a total of 34 genotyped samples: 21 individuals genotyped for 32 microsatellites plus 13 individuals genotyped for 12 microsatellites. We performed PCR amplifications twice and four times for high quality and low quality DNA, respectively, on MyCycler BioRad Thermal Cyclers. Total reaction volume was 10 µl, including 5 µl of the QIAGEN PCR Master Mix, 1 mL of primer mix, and 2 µl of DNA. We screened amplification products on an ABI 3130xl Genetic Analyzer (Applied Biosystems).

### mtDNA analyses

We checked and edited electropherograms by eye and aligned all sequences using GENEIOUS R6. Then, we pooled mtDNA sequences with previously published sequences from *C. niloticus* and *C. suchus* [21]. Since our goal at mtDNA level was to ensure that all samples belong to *C. suchus* and to check for historical genetic differentiation by identifying mtDNA haplotypes, we created an haplotype network to infer genetic relationships using TCS 1.21 [22].

### Microsatellite analyses

Two out of 35 samples that we successfully analyzed exhibited identical genotypes (a fresh sample and a fecal sample from *guelta* Legleyta), thus we excluded one from the dataset. We performed four PCR replicates for each sample (discarding samples that failed to have a good amplification rate) after a maximum likelihood evaluation of error rates (allelic dropout and false alleles) using Pedant 1.0 [23]. We obtained the consensus genotype after comparison of four replicates using Gimlet v 1.3.3 [24], which we used for computing final error rates for each marker and sample. We tested for significant deviations from Hardy–Weinberg equilibrium and presence of linkage disequilibrium within each of the four inferred genetic demes (corresponding to four river sub-basins systems; see results) at each locus for two genetic databases (32 and 12 loci) using ARLEQUIN 3.5.1.3 [25]. We tested for significance using a Markov chain method with 10,000 dememorization steps and 1,000 batches of 10,000 iterations per batch and sequential Bonferroni correction [26] for multiple comparisons. We used ARLEQUIN to assess indices of population genetic diversity (mean number of alleles per locus (*Na*), and observed (*Ho*) and expected (*He*) heterozygosity values) within each geographic groups (sub-basins) identified in population structure analyses (see below) and using the 12 loci dataset to maximize the number of samples. We calculated allelic richness per sub-basin using the rarefaction algorithm implemented in ADZE 1.0 [27] and standardized to the smallest sample size (N = 5). We used GenAlEx 6.5 [28] to estimate the number of private alleles (Npriv) and the Queller and Goodnight [29] statistic *r_xy_* implemented in IDENTIX 1.1 [30] to calculate pairwise relatedness between all individuals. We also estimated the mean and the variance of *r*_xy_ for each river sub-basin and compared to their expected distribution, generated by 1,000 permutations of genotypes among individuals, under the null hypothesis of no relatedness.

We used the Bayesian genotype clustering method implemented in STRUCTURE 2.3.4 [31] to infer the number of genetic demes present within both datasets (32 loci including samples with missing data and 12 loci). We used 10 runs for each value of *K* (number of genetic demes) ranging from 1 to 7, a burn-in period of 1×10^6^ and a run length of 5×10^6^ iterations. We implemented an admixture model with uncorrelated allele frequencies and without prior information on sample population membership. We used Structure Harvester 0.6.93 [32] to estimate the most likely number of *K* by the highest value of log probability of data L(*K*) [33], and the Δ(*K*) [34], and CLUMPP 1.1.2 [35] to align cluster membership coefficients from the 10 replicate cluster analyses from each *K*-value. We calculated pairwise *F*_ST_ values [36] in ARLEQUIN to estimate genetic differentiation among sub-basins and tested significance of pairwise comparisons using an exact test with 1,000 iterations, adjusting *P* - values with the sequential Bonferroni correction.

To identify spatial genetic patterns we also performed multivariate analyses using the spatial Principal Component Analyses (sPCA) [37], and the Discriminant Analysis of Principal Components (DAPC) [38], in the 12 markers dataset. These two methods do not require data to meet Hardy–Weinberg expectations or linkage equilibrium to exist between loci, and are implemented inside the adegenet package [37] for the R software (R Development Core Team, 2008). sPCA investigates cryptic spatial patterns of genetic variability using georeferenced multilocus genotypes. We performed two statistical tests in sPCA to detect the existence of global (patches and clines) and local structure (strong genetic differences between neighbors). Then, we applied DAPC to identify and describe clusters of genetically related individuals. This multivariate method maximizes variation between groups and minimizes the variation within groups. To define the number of K groups, we first used K-means clustering of principal components (ranging from 1 to 7) and a Bayesian Information Criterion (BIC). We specified the actual number of clusters and performed the DAPC function retaining 20 principal components of PCA.

We used BIMR [39] to estimate migration rates of recent gene flow (*m*) between sub-basins. BIMR uses assignment tests to estimate the proportion of genes derived from migrants within the last generation and assuming linkage equilibrium and allowing for deviation from Hardy-Weinberg equilibrium. We used a burn-in period of 1×10^5^ and a run length of 5×10^5^ iterations for each of the 10 replicate runs, and then averaged pairwise migration rates between and within sub-basins across runs.

## Results

### Historical genetic structure

The final mtDNA dataset resulted in a concatenated alignment of 421 bp of the 12 S gene and 878 bp of the ND4 gene for 39 individuals (25 from this study and 14 Genbank sequences). Haplotype network reconstruction using statistical parsimony in TCS resulted in three haplotype networks not connected under the 95% connection probability criteria (Fig. 1). One network contained all *C. suchus* haplotypes and diverged by 53 mutational steps from the second network containing all *C. niloticus* haplotypes, whereas a third network represented by a single haplotype from Malawi differed in 19 mutational steps from the *C. niloticus* main network. Mauritanian *C. suchus* exhibited low genetic diversity (only three haplotypes), with a common haplotype (Mauritania A) widespread across the whole study area (three mountains and main river sub-basins) and a second haplotype (Mauritania B; differing from Mauritania A by a single point mutation) that is restricted to four samples from the Karakoro-Kolimbiné sub-basin (Fig. 1). A third Mauritanian haplotype (Mauritania C, also present in Burkina-Faso), collected 60 km east of our study area, was reported in a previous study [21] and differs by 5 mutational steps from the most widespread Mauritanian haplotype.

### Genetic diversity and effective population size

All loci showed low to moderate polymorphism when considering the entire dataset. The number of alleles per locus ranged from three (CUD68) to seven (CpP302). One locus deviated from Hardy–Weinberg equilibrium at Karakoro-Kolimbiné sub-basin (CpP1409; *P*<0.05 after Bonferroni correction) for the 12 loci dataset. As this deviation occurred only at Karakoro-Kolimbiné and was not observed for the 32 loci dataset, all loci were retained in the analyses. No evidence of linkage disequilibrium was apparent in any pair of loci/population after adjusting the significance level for multiple comparisons. Mean values of allelic dropout and false alleles amplification across loci (12.5% and 0.0%, respectively) and across samples (7.7% and 0.0%) were low, supporting the robustness of the selected loci.

**Figure 2:**
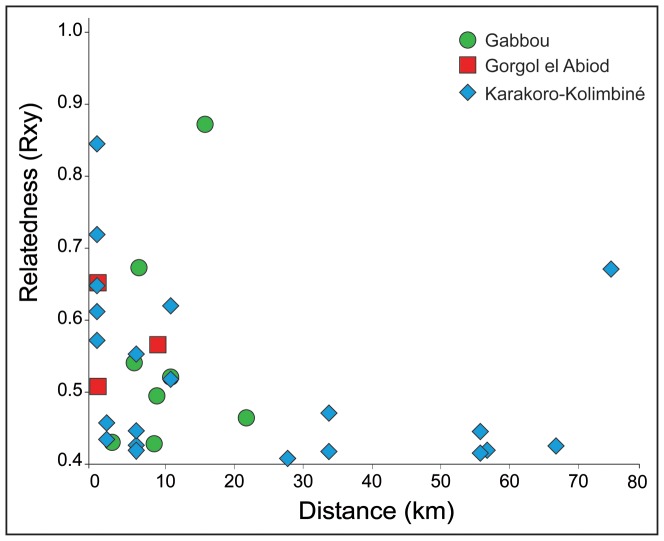
Pairwise relatedness based on Queller & Goodnight (1989) estimator (*r_xy_* higher than 0.4) between individuals of *Crocodylus suchus* across Mauritanian mountains.

Gabbou exhibited the lowest genetic diversity values with the exception of the number of private alleles (Table 1). The highest values of genetic diversity were observed in Gorgol el Akhdar-Garfa for most of the nuclear genetic diversity measures (*A–R*, *Ho* and *He*; Table 1) and in the Karakoro-Kolimbiné sub-basin for the mean number of alleles per locus and number of private alleles (Table 1). Relatedness values were similar among sub-basins (mean *r_xy_* = 0.21–0.25) with the exception of the Gorgol el Akhdar-Garfa sub-basin that showed the lowest level of inbreeding (Table S3). Individuals exhibiting full sibling relationships (*r_xy_*≈0.5) were found within three sub-basins at isolated *gueltas* (Fig. 2; Table S3), although no significant values were achieved for the mean pairwise relatedness within sub-basins.

**Table 1:** Genetic diversity measures for *Crocodylus suchus* on four Mauritanian sub-basins (standard deviation in brackets).

	N	*Na*	*Npriv*	*A*–*R*	*Ho*	*He*
Gabbou	8	3.33 (0.98)	6	2.37 (0.46)	0.47 (0.18)	0.56 (0.15)
Gorgol el Abiod	6	3.58 (1.44)	4	2.59 (0.68)	0.54 (0.16)	0.61 (0.17)
Gorgol el Akhdar-Garfa	4	3.58 (1.08)	7	2.87 (0.59)	0.62 (0.30)	0.74 (0.12)
Karakoro-Kolimbiné	16	4.00 (1.65)	9	2.46 (0.68)	0.54 (0.17)	0.61 (0.14)

Results are based on 12 microsatellite loci. N: number of genotyped individuals; *Na*: mean number of alleles per locus; *Npriv*: number of private alleles; *A*–*R*: allelic richness; *He*: expected heterozygosity; *Ho*: observed heterozygosity.

### Contemporary genetic structure and gene flow

**Figure 3:**
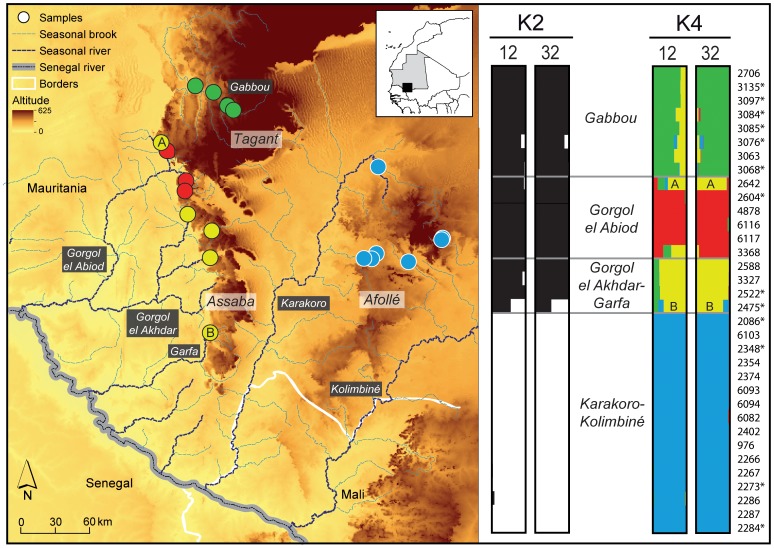
Population structure of *Crocodylus suchus* across Mauritanian mountains. The four genetic demes inferred in STRUCTURE (*K* = 4) are represented with different colors while the two first clusters (*K* = 2) are represented in black and white. Individual assignment to each genetic deme (*K* = 2 and *K* = 4) is shown for each sub-basin (right panel) for 12 and 32 loci. Asterisks indicate non-invasive samples (only 12 microsatellites genotyped) and letters (A and B) represent recent dispersal and historical admixture of the samples 2642 and 2475, respectively.

STRUCTURE analyses identified four genetic demes (*K* = 4) as the most probable number of groups within our dataset (estimated at both L(*K*) and Δ(*K*) methods). These genetic groups correspond to independent hydrographic sub-basins, Gabbou and Gorgol el Abiod in the Tagant mountain, and to combinations of neighbor sub-basins within mountains, Gorgol el Akhdar-Garfa in Assaba and Karakoro-Kolimbiné in Afollé (Fig. 3). The first genetic split (*K* = 2) clearly shows a partition between sub-basins in the Tagant and Assaba mountains (Gabbou, Gorgol el Abiod and Gorgol el Akhdar-Garfa) from sub-basins in the Karakoro-Kolimbiné in the Afollé mountains (Fig. 3). Genetic assignments were similar in both datasets (12 and 32 loci) for each number of imposed clusters (*K*), suggesting that increasing the number of markers slightly improve the resolution of population structure (Fig. 3). One exception occurred with one sample in the Gorgol el Abiod that was assigned to the genetic deme occurring at Gorgol el Akhdar-Garfa. When three clusters are imposed (*K* = 3), sub-basins within Tagant and Assaba mountains are split (results not shown).

**Figure 4:**
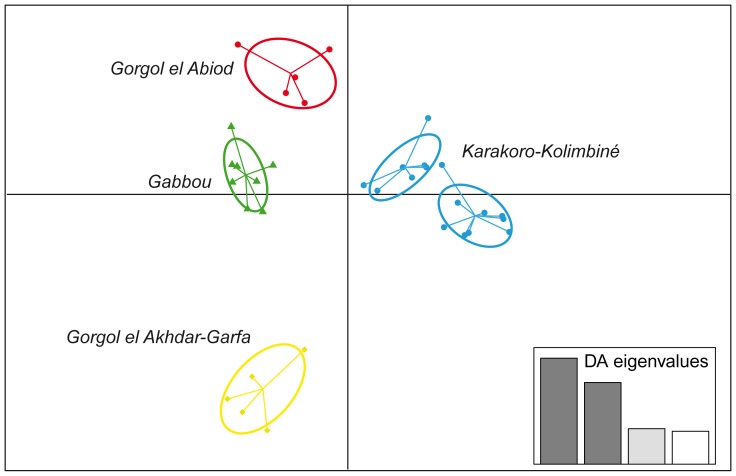
Plot of the first two axes obtained in the Discriminant Analysis of Principal Components (DAPC) using 12 microsatellite data. Dots represent individuals and ellipses roughly group them in five clusters. Color codes identify the same four genetic clusters obtained with STRUCTURE (Fig. 3).

The barplot of the eigenvalues of the sPCA, and the global randomized Monte–Carlo test indicated *global* spatial structure of the sampled genotypes (P = 0.009, 9999 permutations), but no *local* structure (P = 0.25, 9999 permutations) has been detected (Fig. 4). DAPC performed essentially as STRUCTURE, showing K = 2 as the most likely number of clusters (Tagant and Assaba mountains *vs*. Afollé mountains). To investigate deeper genetic structuring in these two groups, we selected the second best number of clusters (K = 5) in which DAPC recovered the four clusters obtained in STRUCTURE and further subdivided the Karakoro-Kolimbiné in two clusters (Fig. 4).

Pairwise *F*_ST_ values yielded moderate to high genetic differentiation among all sub-basins, ranging from 0.12 (between Gabbou and Gorgol el Akhdar-Garfa) to 0.24 (between Gorgol el Abiod and Karakoro-Kolimbiné) (Table 2). Estimates of recent gene flow using BIMr were low between all basin pairs (<0.02%, Table 2) and consistent across runs.

**Table 2:** Genetic distances and gene flow estimations between *Crocodylus suchus* sub-basins.

*F*_ST_/*m*	Gabbou	Gorgol el Abiod	Gorgol el Akhdar-Garfa	Karakoro-Kolimbiné
Gabbou	1 (0)	0.15 (<0.001)	0.12 (0.01)	0.21 (<0.001)
Gorgol el Abiod	0.000081 (0.00051)	1 (0)	0.16 (0.003)	0.24 (<0.001)
Gorgol el Akhdar-Garfa	0.000008 (0.00047)	0.000034 (0.00036)	1 (0)	0.15 (<0.001)
Karakoro-Kolimbiné	0.000063 (0.00051)	0.000052 (0.00038)	0.000026 (0.000058)	1 (0.234)

Above diagonal: pairwise estimates of genetic distance (*F*_ST_; *P*-values in brackets). Below diagonal: recent gene flow (*m*, proportion of alleles derived from migrants in the previous generation; standard deviation in brackets) among sub-basins. On the diagonal: recent gene flow (*m*) within sub-basins (standard deviation in brackets).

## Discussion

### Historical vs. contemporary genetic structure

The presence of mtDNA haplotypes of *C. suchus* in Mauritania is consistent with the recent findings of Hekkala et al. [21] that identify western African crocodiles as *C. suchus*. However, relict populations or individuals of *C. niloticus* present in West Africa cannot be excluded since the sample sizes and geographic areas surveyed are still limited [8], [21]. The lack of genetic structure and low genetic diversity (only two mtDNA haplotypes) reflect historical connectivity among extant populations and high levels of panmixia. As in other water-dependent species occurring across the Sahara, a likely hypothesis to explain the absence of historical genetic structure involves the colonization of Mauritanian mountains from southern ranges during a humid phase of the Sahara [5]. Then, with the emergence of a posterior arid phase in the Mid-Holocene, species retreat to their original range or become locally extinct, with some remaining populations restricted to small mountain areas. We also note that long-lived animals with long generation times, such as crocodiles, are prone to exhibit low levels of mtDNA genetic structure [40], [41], [42], [43], which is partially explained by their low metabolic rates [44], low mutation rates during DNA replication [45], and long longevity [46].

The use of fast evolving markers, which are useful to study fine-scale population structure and contemporary evolutionary forces, revealed high levels of genetic structuring (assignment method) and genetic differentiation (*F*_ST_) across relict populations in Mauritania, suggesting that geographic isolation might have started since the beginning of the arid phase. In addition to recent geographic isolation, we also note that demographic factors and drift [47], might also contribute to increase genetic structure among crocodile populations across the Mauritanian mountains in more recent times, as the extreme low population sizes in this biological system could facilitate genetic differentiation by drift. The high *F*_ST_ values (0–15–0.24) between sub-basins of Afollé and sub-basins from both Tagant and Assaba together with the first split of these geographic groups identified with STRUCTURE, suggest that Afollé population may have experienced a longer isolation from the remaining populations. Genetic differentiation and genetic structure of these populations also reflect ecological constraints on dispersal in *C. suchus*. Pairwise *F*_ST_ values between sub-basins are much higher in comparison to those found in other studies of crocodilians [48]–[52]. This unusual genetic differentiation of desert populations in *C. suchus* can be explained by a combination of geographic isolation, low population sizes and genetic drift. In addition, genetic demes correspond mainly to sub-basins (Gabbou, Gorgol el Abiod, Gorgol el Akhdar-Garfa and Karakoro-Kolimbiné) supporting previously hypothesized metapopulation dynamics in the region [8], [9]. We suggest the presence of four metapopulations that mostly correspond to major sub-basins. Seasonal rivers and brooks that are sparsely distributed in temporal river beds within each sub-basin (see Fig. 1) may connect isolated individuals in *gueltas* and *tâmoûrts* during the rainy seasons, while these water bodies act as refugia to relict populations during the dry seasons when they remain isolated by sandy areas. However, individuals from relatively close localities but different sub-basins (less than 50 km between *gueltas*; see Fig. 1) would be largely maintained geographically and genetically disconnected.

### Dispersal capacity and contemporary gene flow

The results obtained from the combination of genetic markers unveiled the development of a metapopulation system of *C. suchus* within the Mauritanian mountains, which likely began with the beginning of the Sahara drying. While mtDNA indicates historical gene flow and spatial connectivity among these relict populations of *C. suchus*, our results based on microsatellite markers revealed low levels of crocodile movements between sub-basins. The migration rates obtained between sub-basin pairs are much lower than the threshold value (*m* = 0.1) considered for demographic independence [53], [54], suggesting that overland dispersal between basins is rare and each sub-basin has independent dynamics. The high migration estimates, as well as possible full siblings, only found within sub-basins reinforce the hypothesis of a metapopulation dynamic acting mostly within each sub-basin. Demographics and genetic structure is highly influenced by water-level fluctuations in water-dependent species. The present spatial dynamics of this metapopulation system would resemble the source-sink dynamics described for other aquatic organisms in which upstream populations serve as sources and downstream populations as sinks [55]. Such spatial dynamics makes upstream colonization less probable and reduces the likeliness of rescue effect in upstream populations [56]. This would explain the admixed genotype found at southern Assaba Mountain (sample 2475), which could have mixed ancestry from Assaba and Afollé migrants (see *K* = 2; Fig. 3). However, our study does not include crocodiles from the Senegal River, which prevented our investigation of the migration dynamics between the Senegal River population (and its tributaries) and isolated mountain populations. Moreover, we are cautious in our interpretations because this species is a long-lived organism, which could delay the detection of a more contemporary gene flow.

The reduction of connectivity among fragmented populations is negatively associated with migration rates and gene flow, decreasing the colonization capacity and the rescue effect that ultimately impact both genetic diversity and structure [57], [58]. If crocodile populations remain isolated, then they are likely to lose genetic diversity over time and increase their susceptibility to extinction by genetic processes, including inbreeding depression, random fixation of deleterious alleles and loss of adaptive potential [59]. Thus, the persistence of relict populations across the Sahara-Sahel largely relies on individuals' dispersal ability, allowing the so-called rescue effect of reduced groups of isolated individuals. Field surveys have found crocodile carcasses of individuals dispersing between water bodies that likely have occurred during the rainy season when water flows partially connect *gueltas* [8], [60]. Some of the few individuals restricted to these *gueltas* might act as dispersers, colonizing other water bodies by overland dispersals. Our study shows evidence of long dispersal between sub-basins for one individual (sample 2642), which leads us to question why and how do crocodiles disperse. Two main benefits of dispersal are the avoidance of kin competition (to limit inbreeding) and the access to better habitats [61], [62]. However, the dispersal costs in this region must be high since habitats outside *gueltas* are unsuitable and individuals would have to move long distances among *gueltas*, thus risking successful dispersal and eventually their lives. Permanent and temporal rivers are thought to be the main way to disperse for crocodiles, as for other semi-aquatic organisms [63]–[65], but they can use alternative dispersal pathways over land, connecting water bodies and enlarging these metapopulation systems. Although, we have evidences of attempts of crocodile dispersal between water bodies, further work (e.g. combining genetic analysis and telemetry techniques) is needed to confirm if individuals are able to migrate and breed in the sink population, which is crucial to maintaining viable metapopulations. Generalizations of dispersal patterns are complex as these may vary at the inter- and intra-individual level. Inter-individual variability is usually associated with behavioral traits [61], [62], while intra-individual variability can occur due to phenotypic plasticity and ontogenetic shifts during the lifetime of an individual [66], [67]. Dispersal corridors, landscape features that facilitate the movement between ecologically important areas [57], can be crucial for these relict populations as they may allow periodical inputs of new alleles from adjacent populations. This process prevents the loss of genetic diversity and increases the species' potential to adapt genetically to environmental challenges [68].

Overall, our study confirms genetic connectivity among isolated water bodies within main sub-basins for *C. suchus*, highlighting that individual's dispersal abilities help maintain current populations by diminishing the negative effects associated with stochastic events and genetic drift, and thus preventing local extinctions and increasing the populations' recovery in this harsh and mostly unsuitable environment.

### Genetic diversity in desert populations of West African crocodile and conservation implications

The current geographic isolation of individual crocodiles across Mauritanian mountains, where they persist in extremely low population sizes (5 individuals in average), make them vulnerable to demographic and environmental events that may reduce genetic variability. However, our results do not show drastically low genetic diversity for any sub-basin. In fact, heterozygosities in *C. suchus* genetic demes (*He* ranging from 0.56 to 0.70, Table 1) are comparable to those recorded in large populations of the alligator *A. mississippiensis* [49], black caimans, *Melanosuchus niger* [48], the Cuban crocodile, *Crocodylus rhombifer* [50] and the American crocodile, *Crocodylus acutus*, [51], [52]. Water-level fluctuations may drive metapopulation dynamics in water-dependent organisms and shape their genetic diversity patterns [69]. Such a metapopulation dynamic system could counteract the decline of genetic diversity in crocodiles across Mauritanian mountains and reduce the risk of losing adaptive capacity due to stochastic changes [70]. Moreover, we found a low impact of isolation on the reduction of genetic diversity, which may be explained by the buffered effect of intrinsic biological traits, such as long-generation times that may help to curb the loss of genetic diversity [71], [72].

Future efforts should be focus on identifying landscape features shaping genetic structure and distribution of genetic diversity across crocodile populations in the region. Further fieldwork efforts are needed within these mountains along with integrative spatial/genetic analyses, such as those employed in landscape genetics approaches [73]. Identifying both the dispersal patterns and the landscape and environmental constrains to individuals dispersal will help in establishing efficient ecological networks [74] that are needed to protect these fragile desert populations, which may be undergoing specific adaptive processes linked to warming temperatures and populations fragmentation. On the other hand, further studies on the biology, behavior and ecology of *C. suchus* in isolated localities as complements to molecular approaches will contribute to understanding population dynamics and dispersal rates. In addition to the use of remote sensing techniques to quantify water availability patterns [14], GPS-based satellite telemetry are promising techniques that may better estimate dispersal rates and the extent of these events.

To curb local population extinctions it is advisable to increase both the carrying capacity of populations and the facilitation of dispersal among populations. Increasing water quality is needed as many *gueltas* are presently overexploited by humans, resulting in water shortage during the dry season, faecal contamination, excessive eutrophication, and increased activities for excavating pools or pumping water [8], [60]. Dispersal between water bodies may be enhanced through the installation of small-scale dams and water-reservoirs. Increasing awareness of local human populations for the value of relict crocodile populations should be encouraged, as dispersing crocodiles are often killed [8]. This study highlights the importance hydrographic networks to crocodile dispersal along river beds, because they reduce the risk of inbreeding and extinctions in these fragile habitats. Such conservation activities have been already proposed to preserve crocodile populations in the Gabbou [60] but should be extended to the remainder sub-basins where *C. suchus* occur, as these may be considered independent conservation units. Identifying and protecting the species' refugia in temporary freshwater ecosystems can contribute to conservation plans designated to enhance the resilience of freshwater biodiversity [75].

Crocodiles are usually viewed as a conservation flagship species and considered a valuable indicator in ecosystem monitoring and restoration programs [76], [77]. In contrast to other African regions, villagers from Mauritanian mountains peacefully accept crocodile presence in *gueltas* [8], [9], [60], [78] and even consider their potential for local economy by launching crocodile-watching activities [60]. Moreover, *C. suchus* in Mauritania constitutes a good candidate umbrella species, since it's conservation is tied to other aquatic or semi-aquatic organisms (e.g. amphibians, *Amietophrynus xeros*, *Hoplobatrachus occipitalis*; fishes, *Barbus* spp., *Tilapia* spp., *Clarias anguillaris*; reptiles, *Varanus niloticus*, *Python sebae*), which also live in isolated spots across the Sahara-Sahel. The use of genetic tools to study dispersal in umbrella species has been considered as a realistic approach to implement ecological networks that could assist species protection at the regional level [74]. Therefore, our study on *C. suchus* provides an initial framework to manage and protect threatened desert populations that depend on permanent or seasonal water bodies.

## Supporting Information

Table S1 ***Crocodylus suchus*** **samples analysed in this study.** For each sample (ID), we list the country, mountain chain, basin, sub-basin, locality, GPS coordinates of collection sites and their genetic assignment at both mtDNA (haplotype) and microsatellite level (genetic deme).
(PDF)

Table S2 **Loci used on this study with primer information, allele ranges, PCR conditions and references for description of primers used both for mtDNA and microsatellite markers.**
(PDF)

Table S3 **Pairwise relatedness (** ***r_xy_*** **) among all crocodile individuals.**
(PDF)

Input File S1 **Input file (Structure format) containing genotype information for 32 microsatellite markers.**
(TXT)

Input File S2 **Input file (Phylip format) containing sequences of the 12 s mtDNA fragment.**
(TXT)

Input File S3 **Input file (Phylip format) containing sequences of the ND4 mtDNA fragment.**
(TXT)
