# Supplementary material for: Should I Stay or Should I Go? Dispersal and Population Structure in Small, Isolated Desert Populations of West African Crocodiles
Source: PLoS One. 2014 Apr 16;9(4):e94626. doi: 10.1371/journal.pone.0094626 (PMC3989217; doi:10.1371/journal.pone.0094626)
Supplement: Table S1 — Crocodylus suchus samples analysed in this study. For each sample (ID), we list the country, mountain chain, basin, sub-basin, locality, GPS coordinates of collection sites and their genetic assignment at both mtDNA (haplotype) and microsatellite level (genetic deme). (PDF) [file pone.0094626.s001.pdf]

Table S1. *Crocodylus suchus* samples analysed in this study. For each sample (ID), we list the country, mountain chain, basin, sub-basin, locality, GPS coordinates of collection sites and their genetic assignment at both mtDNA (haplotype) and microsatellite level (genetic deme).

ID	Country	Mountain	Basin	Sub-Basin	Local	Latitude	Longitude	mtDNA Hap.
2706	Mauritania	Tagant	Gabbou	Gabbou	Dar-Salam	17,896501	-12,126695	Mauritania-A
**3135***	Mauritania	Tagant	Gabbou	Gabbou	M'cherba, guelta	17,855314	-12,068843	ND
**3097***	Mauritania	Tagant	Gabbou	Gabbou	Kabda	17,846378	-11,962097	ND
3089	Mauritania	Tagant	Gabbou	Gabbou	Erkenate	17,841530	-11,956260	Mauritania-A
**3084***	Mauritania	Tagant	Gabbou	Gabbou	Ch'Bayer, guelta	17,762083	-11,882833	ND
**3085***	Mauritania	Tagant	Gabbou	Gabbou	Ch'Bayer, guelta	17,762083	-11,882833	ND
3077	Mauritania	Tagant	Gabbou	Gabbou	Ch'Bayer, guelta	17,756830	-11,874920	Mauritania-A
3076*	Mauritania	Tagant	Gabbou	Gabbou	Rh' Zembou, guelta	17,741630	-11,873857	Mauritania-A
3078	Mauritania	Tagant	Gabbou	Gabbou	Rh' Zembou, guelta	17,717370	-11,876082	Mauritania-A
3063	Mauritania	Tagant	Gabbou	Gabbou	Amzouzef, guelta	17,706072	-11,826152	Mauritania-A
**3068***	Mauritania	Tagant	Gabbou	Gabbou	Amzouzef, guelta	17,706072	-11,826152	ND
2642	Mauritania	Tagant	Senegal	Gorgol el Abiod	Garaouel, guelta	17,451667	-12,394850	Mauritania-A
**2604***	Mauritania	Tagant	Senegal	Gorgol el Abiod	E-n-Guinâr, guelta	17,401433	-12,364150	ND
3422	Mauritania	Tagant	Senegal	Gorgol el Abiod	E-n-Guinâr, guelta	17,401433	-12,364150	Mauritania-A
4878	Mauritania	Assaba	Senegal	Gorgol el Abiod	Aouînet Nanâga	17,152482	-12,199115	Mauritania-A
6116	Mauritania	Assaba	Senegal	Gorgol el Abiod	Aouînet Nanâga	17,152482	-12,199115	Mauritania-A
**6117**	Mauritania	Assaba	Senegal	Gorgol el Abiod	Aouînet Nanâga	17,152482	-12,199115	ND
3368	Mauritania	Assaba	Koûrourai	Gorgol el Abiod	Oumm Icheglâne	17,070297	-12,207848	Mauritania-A
2588	Mauritania	Assaba	Senegal	Gorgol el Akhdar	Bâfa	16,888725	-12,184868	Mauritania-A
**3326***	Mauritania	Assaba	Senegal	Gorgol el Akhdar	Legleyta, guelta	16,756482	-11,997233	ND
3327	Mauritania	Assaba	Senegal	Gorgol el Akhdar	Legleyta, guelta	16,756482	-11,997233	Mauritania-A
**2522***	Mauritania	Assaba	Senegal	Gorgol el Akhdar	Foum Goussas	16,547455	-12,009590	ND
**2475***	Mauritania	Assaba	Senegal	Garfa	Goumbel, guelta	15,957078	-12,009859	ND
2490	Mauritania	Assaba	Senegal	Garfa	Goumbel, guelta	15,957078	-12,009859	Mauritania-A
**2086***	Mauritania	Afollé	Senegal	Karakoro	Jaraaziza	17,261378	-10,690148	ND
2096	Mauritania	Afollé	Senegal	Karakoro	Taghtâfet, tâmoûrt	17,329165	-10,708572	Mauritania-B
**6103**	Mauritania	Afollé	Senegal	Karakoro	Oumm el Mhâr, guelta	16,579150	-10,704550	ND
**2348***	Mauritania	Afollé	Senegal	Karakoro	Oumm el Mhâr, guelta	16,579150	-10,704550	ND

2354	Mauritania	Afollé	Senegal	Karakoro	Oumm el Mhâr, guelta	16,579150	-10,704550	Mauritania-A
2374	Mauritania	Afollé	Senegal	Karakoro	Oumm el Mhâr, guelta	16,579150	-10,704550	Mauritania-B
**6093**	Mauritania	Afollé	Senegal	Karakoro	Metraoucha, guelta	16,538033	-10,741550	ND
**6094**	Mauritania	Afollé	Senegal	Karakoro	Metraoucha, guelta	16,538033	-10,741550	ND
**6082**	Mauritania	Afollé	Senegal	Karakoro	Bougâri, tâmoûrt	16,540090	-10,801490	ND
2402	Mauritania	Afollé	Senegal	Karakoro	Bougâri, tâmoûrt	16,540090	-10,801490	Mauritania-A
976	Mauritania	Afollé	Senegal	Kolimbiné	Chegg el Mâleh source	16,515562	-10,452908	Mauritania-A
2266	Mauritania	Afollé	Senegal	Kolimbiné	Chegg el Mâleh source	16,515562	-10,452908	Mauritania-A
2267	Mauritania	Afollé	Senegal	Kolimbiné	Chegg el Mâleh source	16,515562	-10,452908	Mauritania-A
**2273***	Mauritania	Afollé	Mefga	Kolimbiné	Kour, tâmoûrt	16,701802	-10,183587	ND
2286	Mauritania	Afollé	Mefga	Kolimbiné	El Mefga, guelta	16,687579	-10,191363	Mauritania-B
2287	Mauritania	Afollé	Mefga	Kolimbiné	El Mefga, guelta	16,687579	-10,191363	Mauritania-B
**2284***	Mauritania	Afollé	Mefga	Kolimbiné	El Mefga, guelta	16,687579	-10,191363	ND
5323	Guinea-Bissau	Bolama	none	none	Uíte, PN Orango	11,170000	-16,090900	Guinea
3632	Malawi	Lake Malawi	none	none	Mumbo island	-13,968201	34,814248	Malawi-B

* non-invasive samples (only 12 microsatellites genotyped)

In italics: samples only sequenced

In bold: samples only genotyped

ND = No data
