# Supplementary material for: Should I Stay or Should I Go? Dispersal and Population Structure in Small, Isolated Desert Populations of West African Crocodiles
Source: PLoS One. 2014 Apr 16;9(4):e94626. doi: 10.1371/journal.pone.0094626 (PMC3989217; doi:10.1371/journal.pone.0094626)
Supplement: Table S2 — Loci used on this study with primer information, allele ranges, PCR conditions and references for description of primers used both for mtDNA and microsatellite markers. (PDF) [file pone.0094626.s002.pdf]

Table S2. Loci used on this study with primer information, allele ranges, PCR conditions and references for description of primers used both for mtDNA and microsatellite markers.

Marker	Locus ID	Primer Sequences (5´- 3´)	Allele range (bp)	PCR conditions	Reference
mtDNA	L1091<br>H1478	F: CTGGGATTAGATACCCCACTAT<br>R: TGAGGAGGGTGACGGGCGGT	421	1	Kocher *et al*., 1989
	ND4<br>Leu	F: CACCTATGACTACCAAAAGCTCATGTAGAA<br>R: CATTACTTTTACTTGGAATTTGCACCA	878	2	Arevalo *et al*., 1994
Microsatellites	Cj16*	F: CATGCAGATTGTTATTCCTGATG<br>R: TGTCATGGTGTCAATTAAACTC	168-188	MP8	FitzSimmons *et al*. 2001
	Cj18	F: ATCCAAATCCCATGAACCTGAGAG<br>R: CCGAGTGCTTACAAGAGGCTGG	223-247	MP2	FitzSimmons *et al*. 2001
	Cj35*	F: GTTTAGAAGTCTCCAAGCCTCTCAG<br>R: CTGGGGCAAGGATTTAACTCTC	166-188	MP8	FitzSimmons *et al*. 2001
	Cj101	F: F ACAGGAGGAATGTCGCATAATTG<br>R: GTTTATACCGTGCCATCCAAGTTAG	379-389	MP2	FitzSimmons *et al*. 2001
	Cj104	F: TCCTTCCATGCATGCACGTGTG<br>R: GTTTCAGTGTCTGGTATTGGAGAAGG	222-226	MP2	FitzSimmons *et al*. 2001
	Cj119*	F: GTTTGCTGTGGAATGTTTCTAC<br>R: CGCTATATGAAACGGTGGCTG	193-207	MP8	FitzSimmons *et al*. 2001
	Cj127	F: CCCATAGTTTCCTGTTACCTG<br>R: GTTTCCCTCTCTGACTTCAGTGTTG	347-411	MP2	FitzSimmons *et al*. 2001
	CUD68*	F: GCTTCAGCAGGGGCTACC<br>R: TGGGGAAACTGCACTTTAGG	154-182	MP8	FitzSimmons *et al*., 2001
	Cj131	F: GTTTGTCTTCTTCCTCCTGTCCCTC<br>R: AAATGCTGACTCCTACGGATGG	242-246	MP2	FitzSimmons *et al*., 2001
	CpDi11*	F: GTTTTGAAATGCCACTAATACAC<br>R: CAGTCGGGCGTCATCATAGAGGCAGATAGACAAGAG	178-190	MP7	Miles *et al*., 2009
	CpDi21	F: CAGTCGGGCGTCATCAAAACAGTTGGCTCTGTG<br>R: GTTTATACTTCCTGTGGCATCAT	189-203	MP1	Miles *et al*., 2009
	CpDi24*	F: GTTTACACAGCCCTAATACACA	133-149	MP5	Miles *et al*., 2009

		R: CAGTCGGGCGTCATCATTTTGAGTGGGGATAATAA			
	CpDi28	F: CAGTCGGGCGTCATCACTATGCACTCCCTGATTTAAG<br>R: GTTTCCCACTCACGAATCTAAAG	116-120	MP1	Miles *et al.*, 2009
	CpDi29*	F: GAAACAGCCAAATGTGAG<br>R: CAGTCGGGCGTCATCAGGTAGCTCCAAGTAGTTTATT	253-263	MP4	Miles *et al.*, 2009
	CpDi42	F: GTTTTTCAGTTTATTTGCCAAAG<br>R: CAGTCGGGCGTCATCAGATTGGGGAGGGAAGT	119-131	MP1	Miles *et al.*, 2009
	CpP106	F: CAGTCGGGCGTCATCATAGAAAGAAATGGGCTAGTGT<br>R: GTTTGTATACCCAGGAAAGATTTTG	242-262	MP3	Miles *et al.*, 2009
	CpP218*	F: GTTTGGCATTTGAATTATTAACT<br>R: CAGTCGGGCGTCATCACTGGCAAATCACTTCTG	178-198	MP6	Miles *et al.*, 2009
	CpP302*	F: GTTTGGAACCCAAGAACTTACAAC<br>R: CAGTCGGGCGTCATCATTGGGTTTAGTCAGCACATA	180-216	MP5	Miles *et al.*, 2009
	CpP305	F: GTTTGTAGCTGGAACCTGATAGTG<br>R: CAGTCGGGCGTCATCAGGTTAACACGTGGTAACTACA	196-246	MP1	Miles *et al.*, 2009
	CpP307	F: CAGTCGGGCGTCATCAGAAACCAGAGGCCAATA<br>R: GTTTCTTGTCTTTGGCAGATT	320-346	MP3	Miles *et al.*, 2009
	CpP309	F: GTTTAATACCTGGCATGTGTTCTTC<br>R: CAGTCGGGCGTCATCACATCAGGTTGGCATTTCA	329-369	MP3	Miles *et al.*, 2009
	CpP722	F: GTTTGAATTGTTTTAGTGTCTGTC<br>R: CAGTCGGGCGTCATCAAGGGTATGCGAGTTTA	119-135	MP3	Miles *et al.*, 2009
	CpP801	F: CAGTCGGGCGTCATCATTGGCATTAGATTGGTAGAC<br>R: GTTTCTATGCCAAAGCTACAAC	167-191	MP1	Miles *et al.*, 2009
	CpP1409*	F: GTTTATGCCCTACTGGTTATCTATC<br>R: CAGTCGGGCGTCATCAGGGAAGGGGATTTAATAAT	242-278	MP4	Miles *et al.*, 2009
	CpP1416	F: GTTTCAAATATATCTTGCCATACA<br>R: CAGTCGGGCGTCATCAGAACAGCGAAAGAACA	188-194	MP1	Miles *et al.*, 2009
	CpP2504	F: CAGTCGGGCGTCATCACTCATATTTCCCAACTATCAC<br>R: GTTTCATTCCCACAATACACATAA	328-346	MP3	Miles *et al.*, 2009
	CpP3004*	F: CAGTCGGGCGTCATCAGGAGTGAATCTATGCCAGC<br>R: GTTTAAAATGTTTTCATATGGTCG	132-164	MP6	Miles *et al.*, 2009

	CpP3309	F: CAGTCGGGCGTCATCATTTATAGGCAAACACAGTC<br>R: GTTTCCCTTTGCATTAATTCTA	154-158	MP3	Miles *et al.*, 2009
	CpP4006	F: CAGTCGGGCGTCATCAAGTGAGATTTGGGTATATTT<br>R: GTTTCATTTCCTTACCATGATAG	103-115	MP3	Miles *et al.*, 2009
	CpP4116*	F: CAGTCGGGCGTCATCATTTCAAATATCCGTGTCAT<br>R: GTTTACCGCTTGAACCTTGT	206-222	MP4	Miles *et al.*, 2009
	CpP4308	F: CAGTCGGGCGTCATCACATATGTAAATTTGGAATGA<br>R: GTTTGATTGAGCCATCCTTAAC	112-127	MP1	Miles *et al.*, 2009
	CpP4311	F: CAGTCGGGCGTCATCAGGCTGCTCTGTGTTTG<br>R: GTTTGGGTTTAGCATCATGT	202-230	MP1	Miles *et al.*, 2009

* Microsatellite loci used for non-invasive samples

References
Arevalo et al. (1994). *Systematic Biology*, **43**, 387-418.
FitzSimmons et al. (2001). In: Crocodilian biology and evolution (ed. by G.Grigg, F.Seebacher and C.E.Franklin). Chipping Norton, Australia: Surrey Beatty; 51-57.
Kocher et al., (1989). *Proc. Natl. Acad. Sci. USA*, **86**, 6196-6200.
Miles et al. (2009). *Conservation Genetics*, **10**, 963-980.

PCR conditions

1

	95ºC	15 minutes	
	95ºC	30 seconds	11 X
↓ 0.5ºC	55ºC	1 minute	
	72ºC	30 seconds	
	95ºC	30 seconds	28 X
	50ºC	1 minute	
	72ºC	30 seconds	
	60ºC	10 minutes	
	12ºC	∞	

2

	95ºC	15 minutes	

	95ºC	30 seconds	11 X
↓ 0.5ºC	52ºC	1 minute	
	72ºC	45 seconds	
	95ºC	30 seconds	28 X
	47ºC	1 minute	
	72ºC	45 seconds	
	60ºC	10 minutes	
	12ºC	∞	

MP1

	95ºC	15 minutes	
	95ºC	30 seconds	11 X
↓ 1ºC	65ºC	30 seconds	
	72ºC	30 seconds	
	95ºC	30 seconds	21 X
	55ºC	30 seconds	
	72 ºC	30 seconds	
	95ºC	30 seconds	8 X
	53ºC	30 seconds	
	72ºC	30 seconds	
	60ºC	30 minutes	
	12ºC	∞	

MP2

	95ºC	15 minutes	
	95ºC	40 seconds	9 X
↓ 0.5ºC	63ºC	1 minute	
	72ºC	1 minute	
	95ºC	40 seconds	24 X
	59ºC	1 minute	

	72 ºC	1 minute	
	95ºC	40 seconds	8 X
	53ºC	1 minute	
	72ºC	1 minute	
	60ºC	30 minutes	
	12ºC	∞	

MP3

	95ºC	15 minutes	
	95ºC	30 seconds	22 X
↓ 0.5ºC	55ºC	30 seconds	
	72ºC	30 seconds	
	95ºC	30 seconds	18 X
	45ºC	30 seconds	
	72 ºC	30 seconds	
	60ºC	30 minutes	
	12ºC	∞	

MP4

	95ºC	15 minutes	
	95ºC	30 seconds	17 X
↓ 0.5ºC	54ºC	45 seconds	
	72ºC	45 seconds	
	95ºC	30 seconds	33 X
	46ºC	45 seconds	
	72 ºC	45 seconds	
	60ºC	10 minutes	
	12ºC	∞	

MP5

	95ºC	15 minutes	
	95ºC	30 seconds	
↓ 0.5ºC	59ºC	45 seconds	13 X
	72ºC	45 seconds	
	95ºC	30 seconds	
	53ºC	45 seconds	37 X
	72 ºC	45 seconds	
	60ºC	10 minutes	
	12ºC	∞	

MP6

	95ºC	15 minutes	
	95ºC	30 seconds	
↓ 0.5ºC	54ºC	45 seconds	20 X
	72ºC	45 seconds	
	95ºC	30 seconds	
	45ºC	45 seconds	30 X
	72 ºC	45 seconds	
	60ºC	10 minutes	
	12ºC	∞	

MP7

	95ºC	15 minutes	
	95ºC	30 seconds	
↓ 0.5ºC	60ºC	45 seconds	11 X
	72ºC	30 seconds	
	95ºC	30 seconds	
	55ºC	45 seconds	39 X
	72 ºC	30 seconds	

	60ºC	10 minutes	
	12ºC	∞	

MP8

	95ºC	15 minutes	
	95ºC	40 seconds	
↓ 0.5ºC	63ºC	1 minute	11 X
	72ºC	45 seconds	
	95ºC	40 seconds	
	59ºC	1 minute	21 X
	72 ºC	45 seconds	
	95ºC	40 seconds	
	53ºC	1 minute	8 X
	72ºC	45 seconds	
	60ºC	20 minutes	
	12ºC	∞	
