# Supplementary material for: Should I Stay or Should I Go? Dispersal and Population Structure in Small, Isolated Desert Populations of West African Crocodiles
Source: PLoS One. 2014 Apr 16;9(4):e94626. doi: 10.1371/journal.pone.0094626 (PMC3989217; doi:10.1371/journal.pone.0094626)
Supplement: Table S3 — Pairwise relatedness ( rxy ) among all crocodile individuals. (PDF) [file pone.0094626.s003.pdf]

Table S3. Pairwise relatedness (r_{xy}) among all crocodile individuals.

		2706	3135	3097	3084	3085	3076	3063	3068	2642	2604	4878	6116	6117	3368	2588	3327	2522	2475	2086	6103	2348	2354	2374	6093	6094	6082	2402	976	2266	2267	2273	2286	2287	2284
Gabbou	2706	*	0.54	0.87	0.01	0.05	0.10	0.29	0.14	0.05	-0.06	0.00	0.05	0.18	0.07	-0.13	0.02	0.05	-0.23	-0.05	-0.05	-0.25	-0.21	-0.36	-0.13	-0.05	-0.19	-0.16	-0.36	-0.18	-0.38	-0.19	0.04	-0.07	-0.15
	3135		*	0.52	0.07	0.46	0.30	0.20	0.36	0.01	-0.09	0.16	0.02	0.14	0.21	-0.22	-0.13	0.08	-0.20	-0.13	-0.18	-0.27	-0.22	-0.17	-0.18	-0.13	-0.13	-0.09	-0.26	-0.35	-0.30	-0.14	-0.06	-0.20	-0.14
	3097			*	0.08	0.03	-0.17	0.12	0.22	0.19	0.13	-0.01	-0.01	0.11	0.13	-0.14	-0.15	0.14	-0.21	0.01	-0.08	-0.27	-0.29	-0.42	-0.14	-0.10	-0.20	-0.16	-0.43	-0.34	-0.40	-0.29	-0.07	-0.20	-0.20
	3084				*	0.34	-0.15	0.50	0.22	-0.12	0.08	-0.14	0.15	-0.20	-0.05	0.02	-0.07	-0.25	0.03	-0.28	-0.24	-0.06	-0.22	-0.29	0.02	-0.13	-0.26	-0.32	-0.30	-0.33	-0.09	-0.28	-0.13	-0.30	-0.29
	3085					*	0.43	0.21	0.42	-0.06	0.02	0.25	-0.24	0.00	0.30	-0.36	-0.34	0.07	-0.09	-0.30	-0.33	-0.22	-0.16	-0.01	0.00	-0.10	-0.28	0.00	-0.13	-0.34	-0.11	-0.11	-0.16	-0.24	-0.11
	3076						*	0.12	0.68	-0.39	-0.40	0.01	-0.29	-0.06	0.09	-0.24	0.18	-0.10	-0.12	-0.14	-0.02	0.43	0.16	0.24	0.16	0.33	0.58	0.24	0.35	-0.01	-0.08	0.19	-0.27	-0.13	-0.04
	3063							*	0.20	0.05	-0.18	-0.05	0.27	-0.02	0.08	-0.02	0.22	-0.06	0.00	-0.08	-0.14	-0.29	-0.35	-0.21	-0.19	-0.22	-0.24	-0.24	-0.32	-0.40	-0.40	-0.22	-0.11	-0.18	-0.22
	3068								*	0.32	-0.16	0.08	-0.16	-0.07	0.16	-0.11	0.03	0.23	-0.16	0.10	0.00	0.15	0.07	-0.10	0.02	0.10	0.13	0.06	-0.27	-0.25	-0.29	-0.21	-0.06	-0.17	-0.08
Gorgol el Abiod	2642									*	0.34	0.22	-0.10	-0.10	0.21	0.10	-0.18	0.21	-0.30	0.07	-0.02	-0.28	-0.15	-0.21	-0.13	-0.22	0.04	-0.25	-0.30	-0.15	-0.38	-0.38	0.17	0.01	-0.05
	2604										*	0.34	0.36	0.24	0.10	-0.21	-0.37	-0.09	-0.05	-0.09	-0.09	-0.31	-0.33	-0.23	-0.08	-0.30	-0.05	-0.21	-0.19	-0.33	-0.14	-0.33	-0.23	-0.24	-0.24
	4878											*	0.23	0.65	0.56	-0.30	-0.28	-0.03	-0.18	-0.17	-0.29	-0.47	-0.40	-0.22	-0.26	-0.38	-0.17	-0.21	-0.33	-0.35	-0.17	-0.19	-0.13	-0.10	-0.10
	6116												*	0.51	-0.18	-0.08	-0.07	-0.31	-0.06	-0.08	-0.14	-0.34	-0.47	-0.21	-0.14	-0.34	-0.13	-0.25	-0.25	-0.37	-0.28	-0.31	-0.33	-0.34	-0.33
	6117													*	0.20	-0.25	-0.20	-0.21	-0.17	-0.14	-0.25	-0.44	-0.47	-0.18	-0.27	-0.32	-0.18	-0.10	-0.33	-0.21	-0.05	-0.09	-0.09	-0.11	-0.11
	3368														*	-0.17	-0.23	0.22	-0.22	-0.08	-0.24	-0.38	-0.36	-0.27	-0.31	-0.38	-0.18	-0.11	-0.19	-0.44	-0.26	-0.14	-0.18	-0.19	-0.19
Gorgol el Akhdar-Garfa	2588															*	0.03	0.22	-0.21	-0.19	-0.33	-0.19	-0.19	-0.31	-0.15	-0.16	-0.21	-0.33	-0.27	-0.11	-0.05	-0.16	0.00	-0.12	-0.22
	3327																*	-0.14	0.24	0.00	0.00	0.11	0.08	-0.11	-0.04	0.11	-0.04	-0.15	-0.14	-0.02	-0.09	-0.14	0.05	0.02	-0.09
	2522																	*	-0.14	-0.06	-0.30	-0.41	-0.24	-0.23	-0.28	-0.19	-0.25	0.06	-0.33	-0.28	-0.21	-0.11	-0.02	-0.18	-0.18
	2475																		*	-0.18	-0.12	0.10	0.23	0.11	0.15	0.05	-0.04	-0.31	-0.03	-0.07	0.15	-0.09	-0.38	-0.29	-0.28
Karakoro-Kolimbiné	2086																			*	0.67	0.26	-0.08	0.07	-0.08	0.00	0.32	0.18	-0.12	-0.06	-0.12	-0.07	0.16	0.24	0.22
	6103																				*	0.61	-0.01	0.03	0.13	0.13	0.62	0.14	0.12	0.17	-0.20	-0.10	0.10	0.27	0.35
	2348																					*	0.38	0.21	0.39	0.43	0.52	0.09	0.37	0.30	0.12	0.18	-0.03	0.10	0.19
	2354																						*	0.56	0.44	0.55	0.03	0.06	0.31	0.40	0.21	0.37	0.22	0.25	0.23
	2374																							*	0.25	0.42	0.19	0.29	0.36	0.17	0.14	0.42	0.15	0.41	0.44
	6093																								*	0.81	0.06	0.12	0.33	-0.06	-0.21	0.01	-0.16	-0.06	0.04
	6094																									*	0.16	0.28	0.29	0.16	-0.05	0.20	0.08	0.17	0.21
	6082																										*	0.18	0.24	0.20	-0.11	-0.23	-0.24	0.03	0.09
	2402																											*	0.23	0.17	0.03	0.30	0.15	0.28	0.42
	976																												*	0.23	0.04	0.11	-0.18	0.02	0.12
	2266																													*	0.65	0.34	0.47	0.54	0.41
	2267																														*	0.37	0.29	0.25	0.09
	2273																															*	0.39	0.46	0.43
	2286																																*	0.72	0.57
	2287																																	*	0.84
	2284																																		*
